# Assessing arid Inland Lake Watershed Area and Vegetation Response to Multiple Temporal Scales of Drought Across the Ebinur Lake Watershed

**DOI:** 10.1038/s41598-020-57898-8

**Published:** 2020-01-28

**Authors:** Junyong Zhang, Jianli Ding, Pengfei Wu, Jiao Tan, Shuai Huang, Dexiong Teng, Xiaoyi Cao, Jingzhe Wang, Wenqian Chen

**Affiliations:** 1 College of Resource and Environment Sciences, Xinjiang University, Urumqi, 830046 China; 2 Key Laboratory of Oasis Ecology of Ministry of Education, Urumqi, 830046 China; 3 Urumqi Meteorological Satellite Ground Station, Urumqi, 830011 China; 4 School of Computer Science, Xinjiang University of Finance & Economics, Urumqi, 830011 China

**Keywords:** Environmental health, Ecosystem ecology

## Abstract

The Ebinur Lake watershed is an important ecological barrier for environmental changes in the Junggar Basin in Xinjiang Uygur Autonomous Region (XUAR). Due to the tremendous changes in the underlying surface environment of the watershed in the past few decades, the watershed has become a typical region of ecological degradation. Drought affects the surface dynamics and characterizes the regional dry and wet environments, while the dynamic variation in lakes and vegetation are indicators of dynamic changes in land surfaces. Thus, a quantitative assessment of the response of lakes and vegetation to drought conditions at multiple temporal scales is critical for assessing the potential impacts of regional climate change on terrestrial ecosystems and ecological restoration. The standardized precipitation evapotranspiration index (SPEI), the spectral water index (NDWI) and the normalized difference vegetation index (NDVI) were used to analyse the evolution of drought, the variation in lake surface area and the sustainable variation in vegetation. Furthermore, we quantitatively evaluated the response patterns of vegetation to droughts of multiple temporal scales (1-, 3-, 6-, 12-, 24-month). The conclusions showed that (1) overall, the area of Ebinur Lake experienced drastic fluctuations, and the lake area has decreased significantly since 2003, with a dynamic area of 817.63 km^2^ in 2003 and 384.60 km^2^ in 2015, and the lake area had shrank severely. (2) The interannual variation of wet and dry changed alternately during the observation period, and persistent drought events occurred from 2006 to 2010 across the Ebinur Lake watershed. (3) The vegetation area of cultivated land expanded continuously across the watershed, and the grassland degraded severely. (4) The changes in lake surface area are significantly correlated with the inflow water volume (correlation coefficient = 0.64, *P* < 0.01). (5) The vegetation of different terrestrial ecosystems exhibited heterogeneous responses to multiple temporal scales of drought in different seasons. The percentage was 72.78% of the total area, which showed a correlation between vegetation and drought conditions during the growing season period, and there were more impacts of drought on vegetation, with values as high as 64.33% of the area in summer, than those in other seasons.

## Introduction

Drought disasters are a natural climate phenomenon that occur when water availability is obviously below normal levels over long periods, and different types include meteorological drought, agricultural drought, hydrological drought and socio-economic drought, which are a popular issue in water resource research in arid regions^1,2^. Previous research over the last few decades has demonstrated that the frequency and intensity of drought have increased significantly, leading to severe threats in relation to water resources, natural ecosystems and food security^3–5^. Long-term meteorological drought will evolve into agricultural and hydrological drought, and drought severity is an important indicator of regional dry and wet environments. Different terrestrial ecosystems have shown different responses to drought, but drought is undoubtedly one of the main factors driving the reduction of landscape aboveground net primary productivity^1^. However, a general theory of the effects of drought on terrestrial ecosystems is lacking due to their inherent complexity and the limited knowledge of the seasonal drought impacts on vegetation productivity over multiple temporal resolutions^6^. Thus, quantifying and predicting the responses of different terrestrial ecosystems to arid climates are a crucial challenge for climate research^1^. One of the major difficulties in evaluating drought is the selection of indicative variables. A single drought indicator cannot accurately assess drought conditions such as drought duration, drought intensity or drought magnitude^1^. Recently, many drought indices have been developed for drought monitoring; these indices include meteorological drought indices (e.g., the Palmer drought severity index (PDSI)^7^, the standardized precipitation index (SPI)^8^ and the standardized precipitation evapotranspiration index (SPEI)^1^. Additional many indices have been developed based on remote sensing drought indices used for soil moisture drought monitoring (e.g., the temperature vegetation dryness index (TVDI)^9^ and multisource data based integrated indices^10,11^, which have been widely utilized to assess drought conditions. The PDSI is based on the soil water balance equation; however, the PDSI is limited to a fixed temporal scale, which cannot be used evaluate the influence of multiple temporal scales on drought^1^. The SPI addresses the limitations of the PDSI in terms of assessing drought conditions and is widely used in global drought research^12,13^; additionally, the SPI can be calculated for multiple temporal scales^14^. However, the SPI calculation process incorporates only precipitation as a single indicator, ignoring the influence of key climatological factors such as temperature and evapotranspiration; ignoring these aspect can affect the intensity and frequency of drought^15^. Additionally, the accuracy verification of drought indices constructed by remote sensing is a large challenge. Therefore, the SPEI was established by Vicenete-Serrano and this index considers both water deficits and surplus conditions at multiple temporal scales^15^; additionally, the SPEI considers the various meteorological factors affecting drought, which could address the limitations of the PDSI and SPI. Some studies have shown that the SPEI is an effective indicator for describing the occurrence of drought events under the background of global warming, especially in semi-arid and arid areas^1,16,17^.

Ebinur Lake is the largest salt lake in the XUAR. Lakes are a crucial surface water resource and an indispensable part of the wetland environment that plays a role in maintaining the balance of arid regional ecological environments^18^. The evolution process of lakes and the changes in the ecological environment caused by lake changes are consequences of global climate change, regional environmental variations and human economic activities^19^. The expansion and shrinkage of the lake can significantly indicate the consequences of the drying and wetting conditions of the regional environment. Previous studies have shown that the ecological environment surrounding the lakes was destroyed at the beginning of the 1950s^20^. In recent decades, the area of Ebinur Lake has severely declined, which has seriously affected the local ecological environment of the region^21,22^. It can be seen that the long-term dynamic monitoring of lake water resources is of great significance for understanding the arid regional hydrological process.

Vegetation, as another indicator of drying and wetting changes in the regional environment, provides a link between the soil and atmosphere and plays an essential role in the exchange of energy on the surface of the Earth; specifically, vegetation affects the carbon cycle, hydrological cycle and regional human activities^23^. Based on reflectance differences in the red and near-infrared band spectra, the normalized difference vegetation index (NDVI), was used as the response indicator of vegetation to climate change, and the NDVI was derived from satellite imagery, which is widely used to assess vegetation degradation, climate change and global vegetation ecosystem health assessments^4,24,25^. Vegetation productivity is influenced by many factors, and climate change is a key control factor affecting vegetation productivity^26^; specifically, drought events, as the most important global climate hazard, may lead to reduced vegetation productivity^5^. Although temperature and sun radiation are necessary for vegetation growth due to their effects on photosynthesis, water transformation in the form of soil moisture is essential for vegetation growth, especially during critical periods^27^. Thus, the water balance is one of the most crucial factors in determining the global vegetation distribution. In arid regions, the distribution of vegetation is highly dependent on water resource availability^28^. Zhao *et al*. evaluated the responses of vegetation to droughts of multiple temporal resolution across China and indicated that vegetation productivity and SPEI were significantly positively correlated in most regions of China^29^. However, different vegetation types may have different response resistances and temporal responses to drought. Recently, the concept of temporal scales has been used to quantitatively analyse the influence of drought on vegetation due to the differences in vegetation types resulting in different responses to drought^30^. Thus, evaluating the relationship between drought conditions and vegetation type response is of practical significance for studying the relationship between regional changes and terrestrial ecosystems.

In recent years, drought monitoring research in arid regions of the Ebinur Lake watershed has received limited attention. In arid and semi-arid fragile ecological regions, lake dynamics and vegetation variation are indicators of regional drought climate changes^31^. However, some studies have focused only on the relationship between lake surface area changes and climate change and human activities, watershed landscape patterns and land use/land cover change (LUCC) and lake water qualities^22,32,33^. However, the response of vegetation to climate change is rare, especially in arid climates. Moreover, the watershed is primarily based on farming and animal husbandry, resulting in the watershed ecosystem being more susceptible to drought events. Due to the complex landscape heterogeneity (mountain-oasis-desert ecosystem) of the watershed and the different resistance values of vegetation species to drought, there is currently no quantitative analysis for assessing the impact of drought on vegetation. Therefore, reviewing previous studies on the effects of climate on lake area and vegetation dynamics, the link between drought and vegetation growth and changes in inland lake area may help us further understand the effects of drought. In this context, this research attempts to fill in the gaps in the effects of drought climate on regional ecological environments, vegetation and lake dynamics in arid regions. The aim of this paper is to clarify the following points: 1) identify the dynamic evolution of the surface of arid inland salt lakes over the past 16 years; 2) analyse the evolution process of droughts at multiple temporal scales; and 3) reveal the seasonal response sensitivity of different terrestrial ecosystems to drought at multiple temporal scales (1, 3, 6, 12, 24 months).

## Results

### Accuracy evaluation and verification

**Figure 1:**
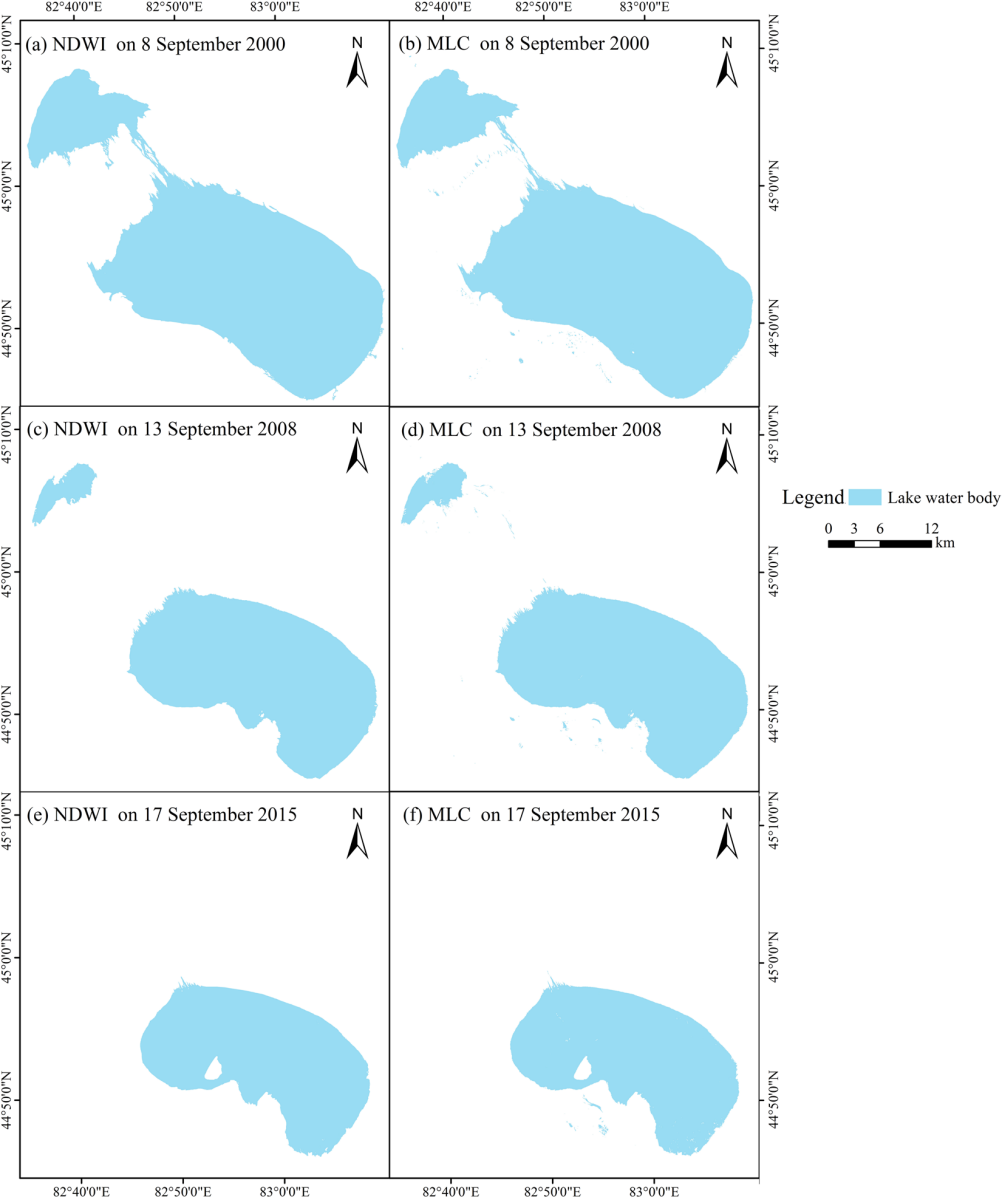
Lake water classification accuracy comparison between the MLC and NDWI.

To evaluate the classification accuracy of the NDWI, in this paper, the maximum likelihood classification (MLC) method based on Landsat-5,7,8 was applied for water body extraction, and the result indicated that the region of interest (ROI) separability values of water and non-water were all greater than 1.99, which can be used for accuracy verification by establishing an error matrix for the NDWI. Compared to the MLC results, the NDWI yields a better overall accuracy in the study region. From a quantitative perspective, the overall accuracy and kappa coefficient of the NDWI for lake water extraction were 99.94% and 0.99, respectively, on September 17th, 2015, which can be applied for the dynamic detection of the Ebinur Lake water surface area with ideal accuracy (Table 1 and Fig. 1).

**Table 1:** Confusion matrix for the water extraction accuracy evaluation using different methods on September 8^th^, 2000, September 13^th^, 2008 and September 17^th^, 2015.

Date	Method	O.A. (%)	Kappa Coefficient	P.A. (%)	U.A. (%)	Commission (%)	Omission (%)
September 7^th^, 2000	MLC	97.74	0.95	94.93	95.46	2.54	1.07
	NDWI	98.85	0.98	96.37	95.89	1.07	0.76
September 13^th^, 2008	MLC	98.83	0.97	96.54	97.35	1.45	0.76
	NDWI	99.17	0.99	97.31	96.68	0.83	0.46
September 17^th^, 2015	MLC	99.25	0.99	98.72	98.64	0.62	0.35
	NDWI	99.84	0.99	99.53	99.68	0.22	0.17

Note: O.A. means overall accuracy; P.A. means producer′s accuracy; and U.A. means user's accuracy.

### Dynamic variation detection of lake surface area

**Figure 2:**
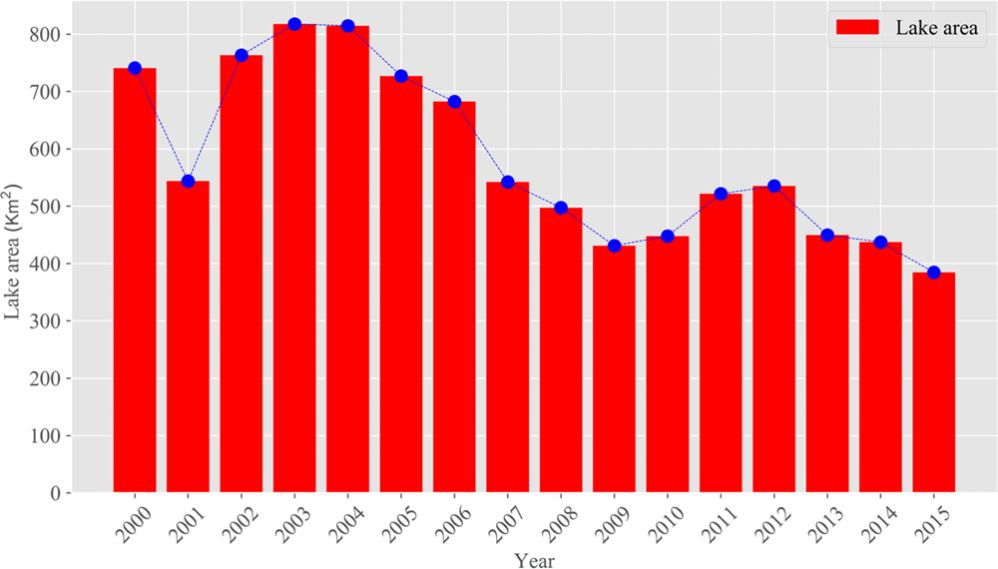
The variation of the Ebinur Lake surface area during 2000 to 2015.

**Figure 3:**
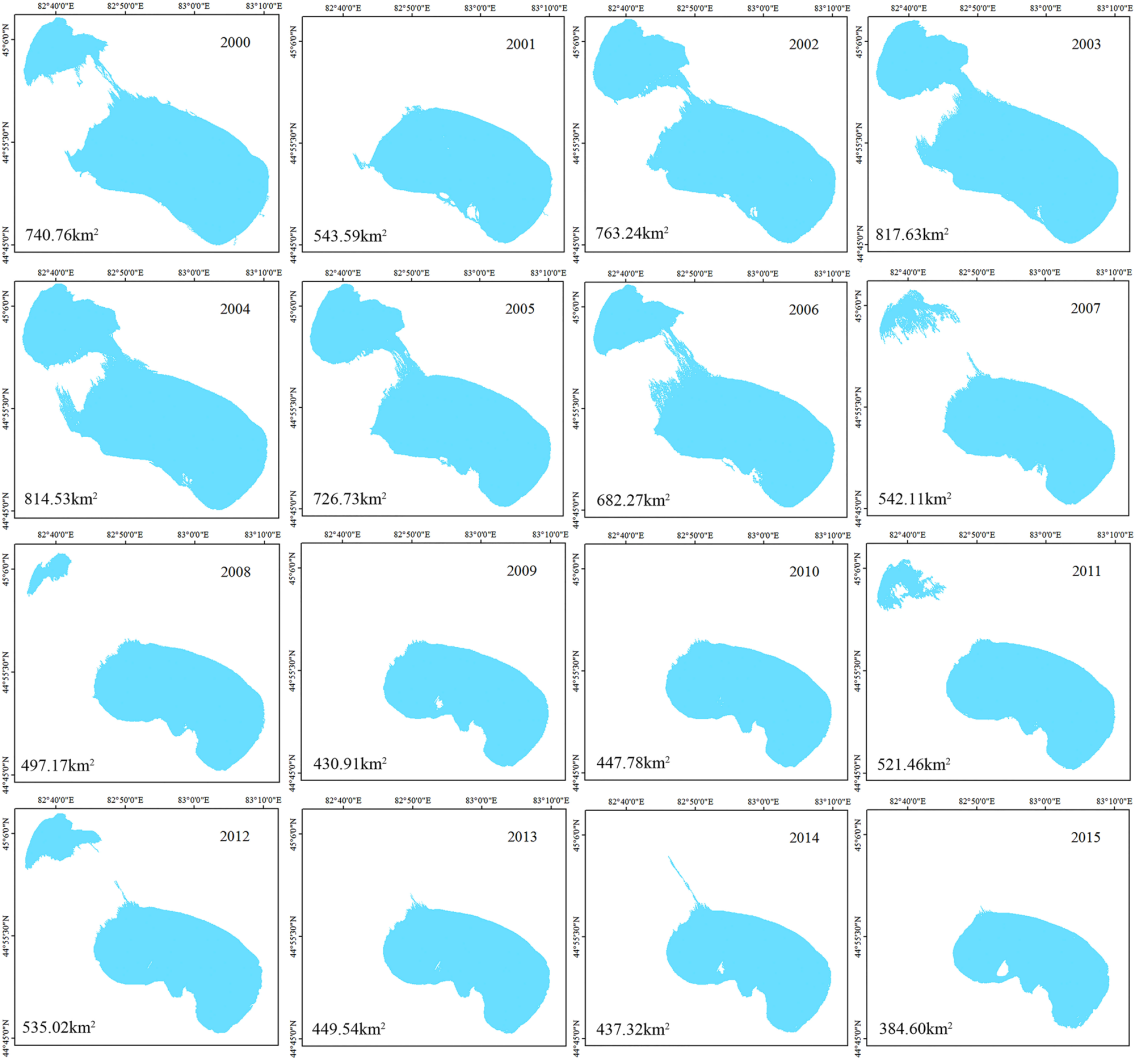
The spatiotemporal variation in the Ebinur Lake level from 2000 to 2015.

Changes in lake water surface areas are essential indicators of different environmental climate conditions at multiple time scales, and the inter-annul variations reflect the responses to climate change^34,35^. Anthropogenic activities have altered the climate and led to changes in the water cycle, especially in fragile arid inland river watersheds^36^. Detecting the inter-annual variation of lake water surface areas is essential to understanding the local hydrological cycle. During the observation period from 2000 to 2015, the inter-annual fluctuation of lake water area changed drastically, and the lake surface area was reduced by 356.16 km^2^. Compared to other years, from 2000 to 2009, the minimum lake water surface area appeared in 2009, which may have been caused by the extreme drought that occurred in the previous year. As can be shown, during the observation period, four peak points appeared in 2003, 2009, 2012 and 2015 (Fig. 2). From 2001 to 2003, the lake surface area increased by 50.04%, with a dynamic change rate of 16.80%. However, the lake surface area shrank by nearly half from 2003 to 2009, with only 430.91 km^2^ in 2009 and a dynamic change rate of −6.73%. From 2009 to 2012, the lake surface areas recovered slightly and changed from shrinking to expanding, with a dynamic rate of 6.04%, which corresponded to an increase of 104.12 km^2^. However, after 2012, the lake surface area decreased sharply, reaching a minimum of 384.60 km^2^ in 2015, with a dynamic change rate of −7.03%. Changes in arid climates, especially in terms of the variation in precipitation and runoff, result in the dramatic fluctuation of lake surface area. During the observation period, the volume of precipitation reached a maximum peak value of 258.53 mm in 2002, and the minimum volume of precipitation was 135.56 mm in 2008. Meanwhile, the SPEI value illustrated that extreme drought events appeared in 2008. The lake surface area severely shrank in 2009. The lake surface area was continuously declining from 2003 to 2010 but began to recover in 2011. Overall, the dynamic variation in the lake surface area is negative. The peak trends in precipitation match fairly well with the peaks at the lake level, though there is a slight delay. From the view of the entire period, the area of Ebinur Lake has deteriorated and is experiencing the processes of expansion and declines (Figs. 2 and 3).

### Trends in drought climate change

**Figure 4:**
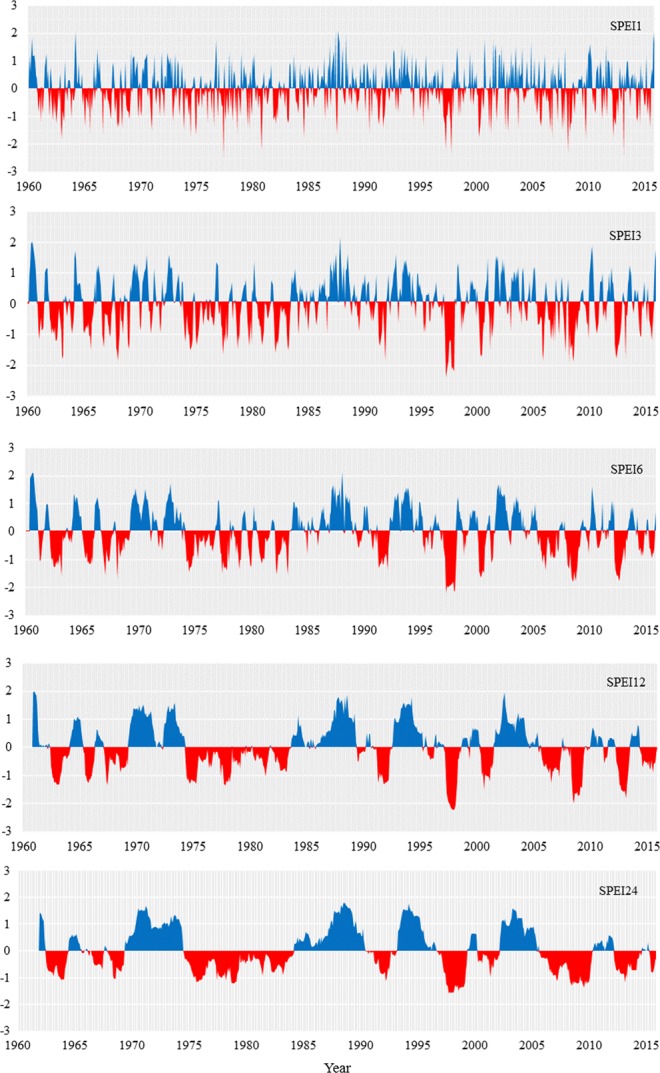
Temporal variation of the SPEI at the 1, 3, 6, 12, and 24-month scale across the Ebinur Lake watershed. The red colour represents the drying period, and the relatively blue colour represents the wetting period.

Research on droughts at multiple temporal scales could characterize meteorological, agricultural and hydrological droughts. Therefore, we calculated the multiple time scale drought index of SPEI-1, SPEI-3, SPEI-6, SPEI-12 and SPEI-24 based on meteorological data. By comparing the SPEI values from multiple temporal scales, the result shows the drought differences at the different time scales (Fig. 4). The 1-month SPEI implied a short-term water surplus and deficit condition. The 3-month SPEI showed an obvious seasonal drought condition. Previous studies have illustrated that the evolution of drought at a 3-month temporal scale with moisture conditions provides obvious climatic balance, which might imply the amount of available moisture in a region^37^. The result showed that alternating wet and drought evolution was found during in the 1960s, and there was consistency in the different time scales (Fig. 4). From the early 1970s to the middle of 1974, the entire watershed was in a wetting period; however, after 1974, a continuous drought occurred for more than a decade. In addition, drought ended and a wetting period began during the middle of the 1980s until the early 1990s. As can be shown, the annual trend of the 12-SPEI showed an obvious reversal in the middle of 1974, and this reversal demonstrated the transition from the wetting trend during the period of 1970–1974 to the drought year period that occurred from 1974 to 1984 (Fig. 4). The peak value of the drought index indicated that extreme droughts occurred in 1997 and 2008 at different temporal scales, with a value up to −2.22 at the 12-month time scale in 1997 and a value of −1.98 in 2008. These results were highly consistent with those of historical drought, which showed that the 12-month or long-term timescales of the SPEI were more appropriate for monitoring inter-annual droughts.

### Spatiotemporal characteristics of the vegetation in the ebinur lake watershed

**Figure 5:**
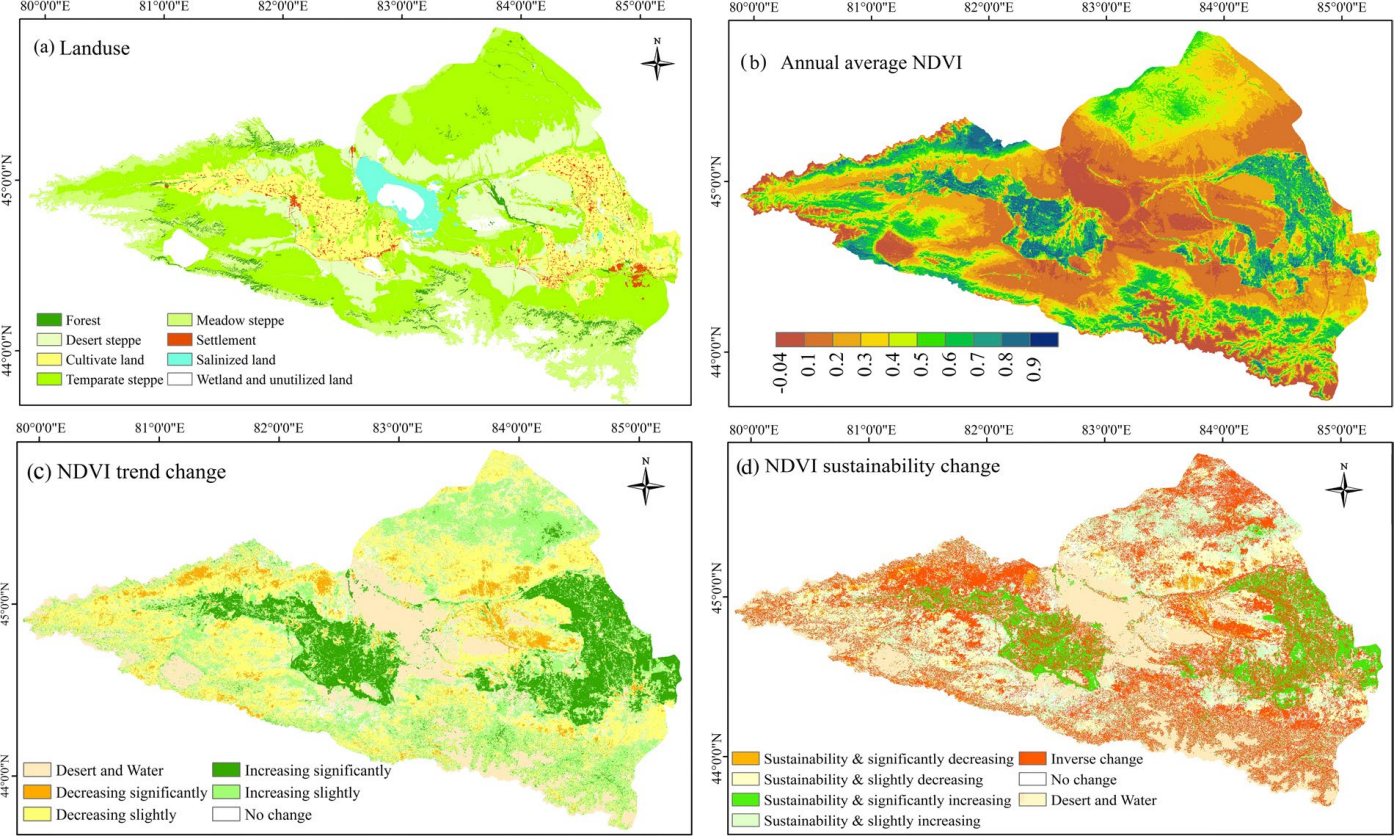
The distribution of terrestrial ecosystem (**a**); annual average NDVI across the Ebinur Lake watershed (**b**); Theil-Sen and Mann-Kendall trends of the NDVI (**c**); sustainable variation in the NDVI from 20001 to 2015 (**d**).

Using the statistical pixel method from the LUCC (Fig. 5a), the grassland area accounts for 62.58% of the entire watershed. As can be shown that the spatial distribution of the NDVI was significantly different. High NDVI values were mainly distributed in mountainous areas and cultivated land, while lower NDVI values were distributed in the central regions with desertification, salinized land surfaces and alpine glaciers (Fig. 5b). The NDVI variation in the Ebinur Lake area can be effectively characterized using the method of Theil-Sen median analysis and the Mann-Kendall test to reveal the temporal and spatial distributions. The characteristics of the NDVI exhibit significant increases in the central oasis area and in the western regions of the whole Ebinur Lake watershed during the observation period. The number of pixels with increasing NDVI trends was 39.74% of the total number of pixels, of which 34.85% of the pixels had significant increases according to the statistical analysis of the pixels (Fig. 5c). This result indicates that increasing pixels are always found in oasis zones and grasslands located in the northern area of Ebinur Lake. However, the number of pixels accounted for 38.07% of all pixels that had decreasing trends, of which 10.52% of the pixels revealed a significant decrease. Compared with the increasing trend area, the pixels that decreased could be found in the north-eastern area of the basin and in the oasis-desert interface of Ebinur Lake. To determine the variation trend and the sustainability of the NDVI in the Ebinur Lake watershed, the database of the trend analysis and the Mann-Kendall trend test was overlaid with the Hurst index results to reveal the sustainability characteristic information of the NDVI. The resultsindicates that continuous growth of the interior oasis had inverse trends, especially in the eastern and northern regions of the Ebinur Lake basin. It can be seen from the figure that the number of pixels with a sustainable decreasing trend of the NDVI was 21.58% of the total number of pixels. Compared with the deceasing trends, the number of pixels with sustainable increasing trends accounted for 22.63% of all pixels. Most of the region's area, i.e., 33.61% of the total number of pixels, had conditions in which the trend developed in the opposite direction. The grasslands in the north-western, northern and south-eastern regions fluctuated severely in the opposite direction (Fig. 5d).

## Discussion

### Lake dynamics and hydrological runoff analysis

**Figure 6:**
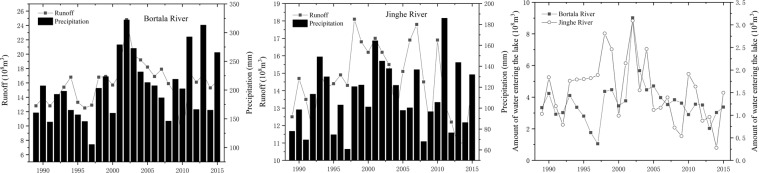
The annual runoff volume, the precipitation and the amount of water entering the lake from the Bortala and Jinghe rivers.

The land surface processes of inland lake dynamics and vegetation production are both indicators of regional climate change. Changes in lake surface area are essential indicators of different environmental conditions at multiple temporal scales^35^. Especially in inland river watersheds, the expansions and reductions of lakes are important indicators of the regional dry or wet environments. The dynamic variation in lake surface area is mainly affected by the comprehensive factors of regional precipitation, temperature, evaporation and inflowing runoff water to the lake, and these factors are of great significance to the local hydrological cycle^36,38^. Some studies have laid the foundation for the research mechanism of the change in lake surface area in the region^39^. Long-term meteorological drought could lead to agricultural and hydrological drought^40^. To further quantify the driving force of integrated elements on lake water surface changes, the meteorological drought index was calculated to characterize the regional dry and wet conditions for exploring the changes in lakes under a certain climate and hydrological background. As can be seen that the lake surface area is consistent with the water flow of the Bortala River and the runoff inflow volume in the same period (Fig. 6). Compared to the Bortala River, the runoff volume of the Jinghe River was lower and consistent with the amount of precipitation, which reached a minimum in 2008. During the observation period, extreme drought events occurred in 2008. The result indicates that the period from 2006 to 2010 is obvious dry at different temporal scales of drought; correspondingly, the lake area consistently declined during this period (Fig. 4). The correlations between total runoff and tributary runoff and lake surface area were calculated separately. The correlation between lake water area and inflow water was 0.64 (r = 0.64, *P* < 0.01) in the Bortala River; however, there was a lower correlation between lake surface area and inflow water in the Jinghe River, which had a value of 0.36 (r = 0.36, *P* > 0.05), indicating that the change in lake surface area was significantly correlated with the annual influx in Bortala. In contrast with the Bortala River, because the northern portion of the Tian Shan is a vital ribbon of economic development in the XUAR^41^, the reason the runoff of the Jinghe River had unrelated flow was caused by the anthropogenic activities, e.g., the available Jinghe urban water and the large amount of irrigation water^32,42^. In addition, the continuous drought from 2006 to 2010 caused the continuous declines in the runoff of the Bortala and Jinghe rivers shown in Figs. 4 and 6, respectively. Moreover, in arid regions, groundwater may hinder the surface runoff from rivers to lakes^43^.

### Spatial distribution of *R*_max_ in different seasons at multiple temporal scales of drought

Climate change, water resource and vegetation productivity are crucial restrictions affecting sustainable social and economic development in the arid region of Northwest China. In addition, the ecosystem vulnerability in these regions has been sensitive to severe changes in climate, causing the evolution of a long-term warming-wetting trend in the last 50 years; furthermore, this trend has been one of decreasing precipitation and increasing evaporation and temperature^39^.

**Figure 7:**
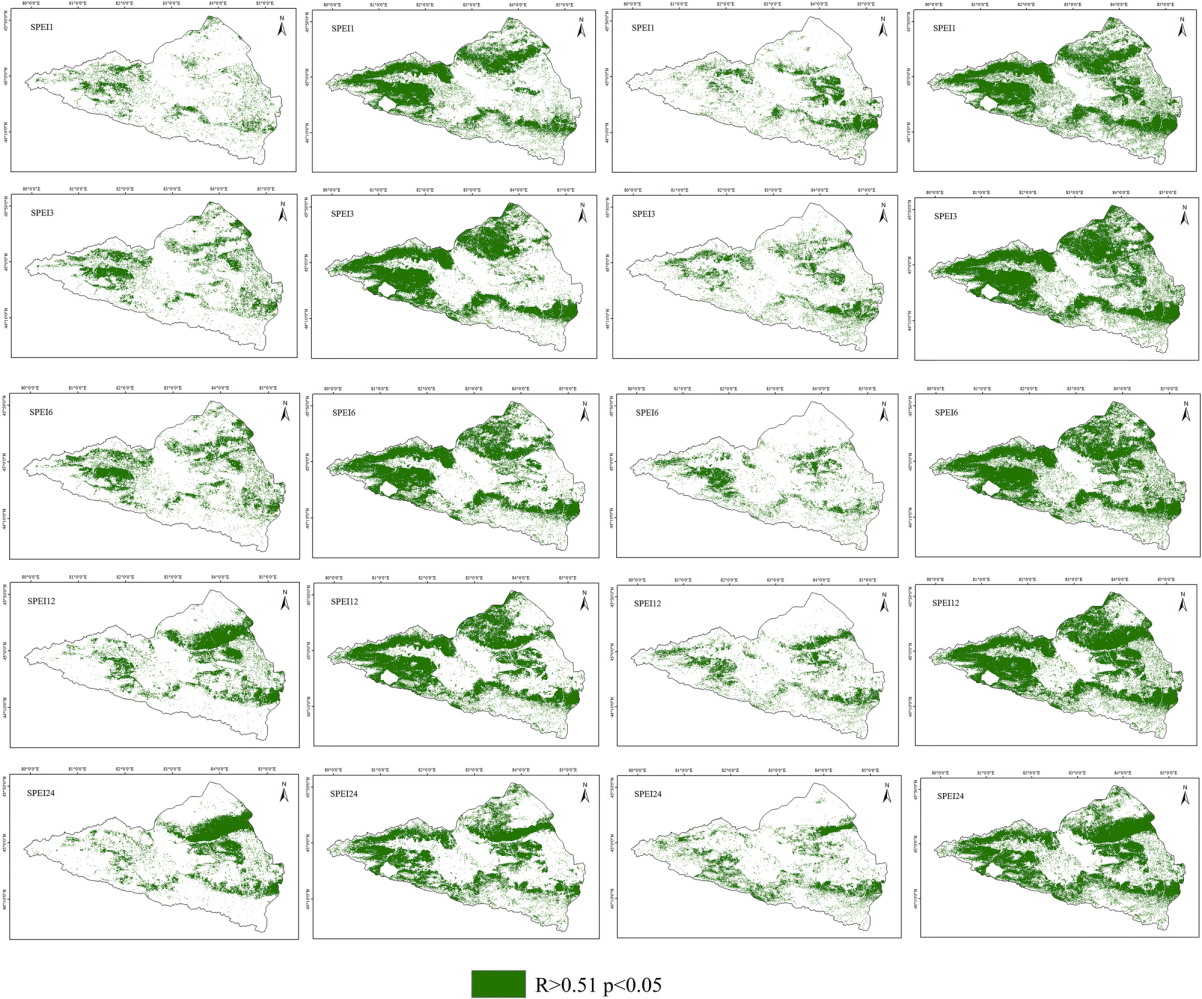
Distribution of significant positive correlations between multiple time-scale SPEI values (1-, 3-, 6-, 12- and 24 month) and seasonal NDVI. A non-significant pattern was shown by the white colour.

To evaluate the response patterns of vegetation to drought in the different vegetation types across the entire Ebinur Lake watershed, the drought severity was quantified at multiple temporal scales using the SPEI in this paper^44^. The results indicate there was a significant response in the variation of vegetation productivity to the multiple temporal scales in different seasons. Similarly, the phenomena of this study confirm that drought monitoring at different temporal scales results in a response of vegetation productivity to drought that is highly heterogeneous, depending on the temporal scales used for drought monitoring; other studies have verified these conclusions^45^. As a major limiting factor of vegetation growing in arid regions, given the different resistance of vegetation types to water deficits, previous studies have analysed the effects of drought on different temporal scales to reflect the sensitivity of vegetation to drought^46^. Based on the analysis of the multiple temporal scales of drought performance of different vegetation types in different growing seasons (spring, summer, autumn and the entire growing seasons), this paper studies the resistance of different vegetation types to multiple temporal scales of drought. The results reported that the maximum correlation coefficient between the SPEI and NDVI reflected the seasonal responses of vegetation activity under drought conditions. However, the maximum correlation coefficient between a shorter time scale for the SPEI and NDVI indicated sensitive responses to drought; additionally, the vegetation responded quickly to changes in soil water content^47^. Compared to short-term scale droughts, the maximum correlation coefficient in the longer time-scale between the SPEI and NDVI indicated that the vegetation had a stronger resistance and resilience to drought effects^1^. Figure 7 demonstrates the distribution of the vegetation response to multiple temporal scales of drought and shows the Rmax index values in the spring, summer, autumn and the entire growing season. In this study, during the entire growing season, the areas with a significant positive correlation between the NDVI and SPEI accounted for 51.55%, 56.54%, 54.47%, 56.71% and 50.47% at the 1-, 3-, 6-, 12-, and 24-month scales, respectively (Fig. 7). It can be seen that the main vegetation types associated with droughts at multiple temporal scales are grasslands, especially during short-term droughts, and desert grasslands, which have a longer response period. Vicente-Serrano reported on the differences in the response patterns of vegetation growth activities to drought indices at multiple temporal scales^1^. Overall, difference responses to drought could be described by the different resistance capabilities of vegetation types to water deficits^48^. However, there is no significant correlation with cultivated area for all droughts at all temporal scales. This result can be explained clearly: during the growing seasons, agricultural crops are mostly located in the oasis plain farming area due to the agricultural irrigation that affects the available water content of soil for vegetation. For different seasons, there are obvious differences in the correlation between the NDVI and SPEI at different temporal scales. In spring, the NDVI was significantly and positively correlated (*P* < 0.05) with multiple temporal scales of the SPEI index, with values of approximately 11.23%, 21.5%, 22.25%, 22.31% and 21.48% of the region, respectively. As seen, there is a significant lag in terms of the vegetation response to drought; however, there are also obvious differences among different vegetation types. Compared to the 1-, 3-, and 6-month drought scales, where the vegetation type is desert steppe, there is a high correlation with the long temporal scale drought index. In the summer, the areas with a significant positive correlation accounted for 38.68%, 44.15%, 43.01%, 46.62% and 38.43% of the study area, respectively (Fig. 7). Compared to spring and autumn, in summer, almost all natural vegetation types had a higher correlation between the NDVI and SPEI at the different temporal scales.

**Figure 8:**
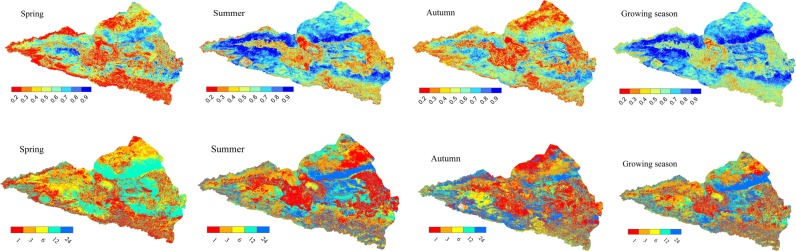
The seasonal pattern of the maximum correlation coefficient between the vegetation and SPEI and the time scales at which the maximum correlation coefficient was obtained across the Ebinur Lake watershed.

To analyse the highest correlation between the NDVI and seasonal drought at multiple temporal scales, we extracted the maximum correlation coefficient (*R*_*max*_) from April to October for the multiple temporal scales (i.e., 1-, 3-, 6-, 12- and 24-month). Figure 8 illustrates that the impact of drought on vegetation changes, and there are obvious differences with season and vegetation type. The *R*_*max*_ of desert steppe vegetation and the SPEI are significantly positive in spring, summer, autumn and the entire growing season, However, the temperate grassland was sensitive to the short-term water deficits of the 3-month and 6-month drought scales, especially in the eastern and northern areas of the watershed, which are mainly covered by grassland vegetation^49^. In the growing season, the NDVI is correlated with drought throughout almost all regions of the Ebinur Lake watershed, including 72.78% of the area. Ji and Peters proposed that the short-term drought variations reflected the changes in soil moisture at the seasonal scale, indicating that the vegetation in the area described above was greatly affected by seasonal precipitation and soil moisture changes^50^. As obviously shown in the woodlands in the Tianshan Mountains, the desert steppe in the northern region and the alpine meadow steppe on the northern slope of the Tianshan Mountains had a significant long-term response to drought that was a long as 12–24 months. In addition to the effects of rainfall, the soil water supply in the high-altitude snowmelt runoff and the freezing of frozen soil weakens the impact of drought on mountain vegetation^49^. As seen from the Fig. 8, the influence of drought on vegetation is stronger in summer than in spring and autumn, with values of 36.86%, 64.33% and 38.61% of the entire area affected by bare land, water, settlement and salinization of the watershed in spring, summer and autumn, respectively (Fig. 8). However, the desert steppe was correlated with the long-term 12-month and 24-month SPEI values due to the physiological characteristics of vegetation, which adjusted its survival strategy to adapt to the effects of drought^17^. It can be found the correlation between the NDVI and SPEI on cultivated land is weak in the area of the Jinghe and Wusu oasis, where human activities are significantly frequent and there are better irrigation conditions, especially in the summer. Due to the complex spatial heterogeneity of the watershed, the influence of topography and agricultural irrigation should be considered when assessing the responses of vegetation to the multiple temporal scales of drought in future research^51^.

**Figure 9:**
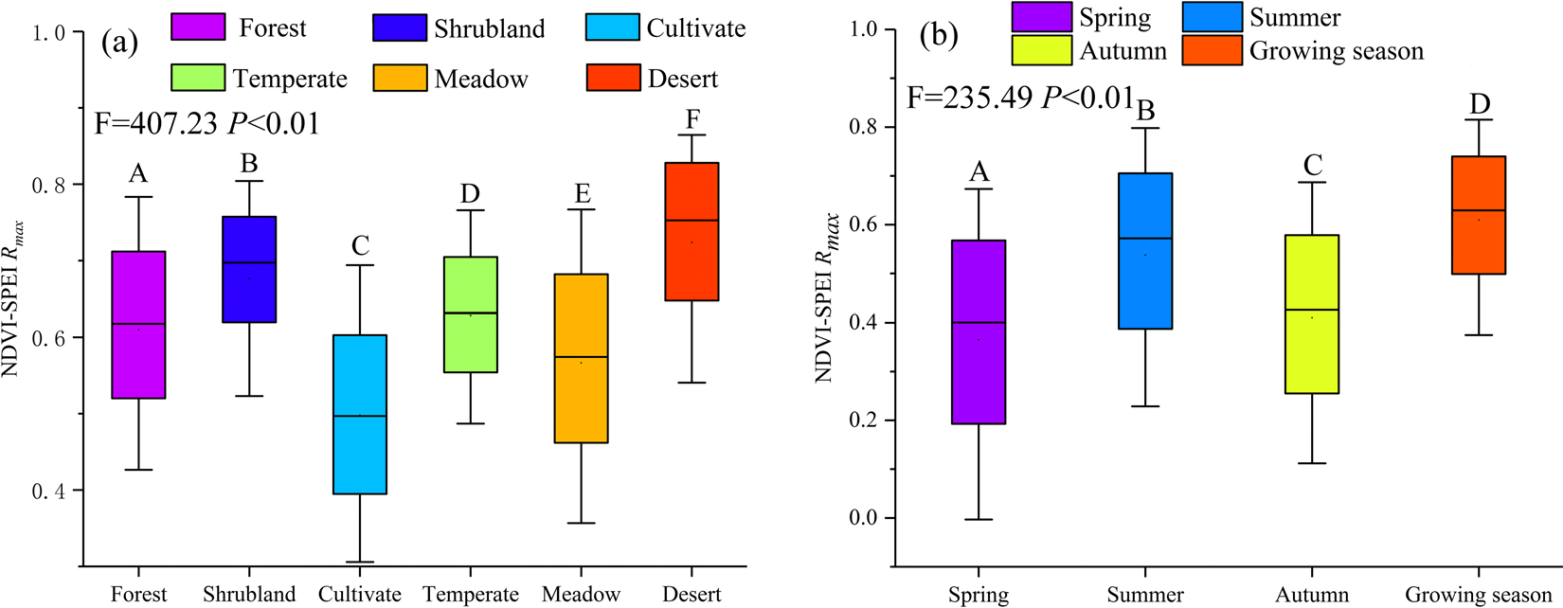
The correlation *R*_*max*_ between the vegetation and SPEI in different seasons in different terrestrial ecosystem types. Note: A, B, C, D, E, F separately represent differences of the Rmax coefficient between the NDVI and SPEI at different levels.

Different correlations and seasonal variations were found in the diverse biome types (Fig. 9). The *R*_*max*_ coefficient between the NDVI and SPEI was higher in the growing season (median, 0.63) and summer (median, 0.57); however, it was lowest in spring (median, 0.4). Moreover, the *R*_*max*_ was higher for desert steppe (median, 0.75) and shrubland (median, 0.7), but it was lower for cultivated land (median, 0.49) and meadow steppe (median, 0.63). Although the correlation between the vegetation and SPEI was mostly positive, different terrestrial ecosystems had significant differences.

### Limitation of the current study

In addition, the distribution pattern of vegetation is constrained by the hydrothermal equilibrium conditions, which means that surpluses and deficits of water resources and vegetation ecosystems play an important role in regulating the terrestrial ecosystems, hydrological processes and socio-economic aspects of the basin. However, both climate change and human activities are closely related to vegetation productivity and lake dynamics. In this paper, only climatic factors were used to analyse the dynamic variation in lake surface area and vegetation production. However, the evapotranspiration process is very complicated and is affected by many factors. Second, drought evolution shows certain spatial and temporal structural dynamics. Therefore, based on this research, multiple factors should be considered comprehensively, and more longer-term and larger spatial scales should be conducted in future research; for example, climate and anthropic activities should be considered in future research.

## Conclusions

In this manuscript, the multiple temporal scales drought indices were calculated to assess the wetting and drying evolution, the dynamic variation of lake surface area and response pattern of vegetation to drought conditions in the watershed; This study provided an sensitivity assessment of different terrestrial ecosystems to multiple temporal scales drought. And the main summary of the results as follows:

1. The interannual variation of wet and dry changed alternately during the observation period;and the results indicated that persistent drought events occurred from 2006 to 2010 across the Ebinur Lake watershed.
2. The conclusion of the dynamic lake variation showed that the correlation between the lake surface area and inflow water volume was 0.64 (r = 0.64, *P* < 0.01), which indicated that the changes in the water surface area were significantly correlated with the inflow water volume. The lake area has decreased significantly since 2003, with a dynamic area of 817.63 km^2^ in 2003 and 384.60 km^2^ in 2015. The overall NDVI of the watershed degraded continously, espercially the grassland condition severely degraded.
3. Overall, we used the maximum correlation coefficient between the NDVI and multiple temporal drought conditions to evaluate the response patterns of different arid terrestrial ecosystems to seasonal drought. The influence of drought on vegetation usually depends on differences in drought resistance and drought stress levels. Among the different terrestrial ecosystems, the temperate grassland showed a high correlation in the short-term (3-,6-months) temporal scale during the growing seasons;and the desert ecological system and alpine meadow grassland showed long-term response period in growing seasons, also revealed siginificant difference in different seasons, which was associated with the resistance of arid vegetation to drought and included certain vegetation survival strategies and features. The results provided crucial information on the responses of different terrestrial ecosystems and lake hydrology to multiple temporal scales of drought in the Ebinur Lake watershed.

## Materials and Methods

### Study area

**Figure 10:**
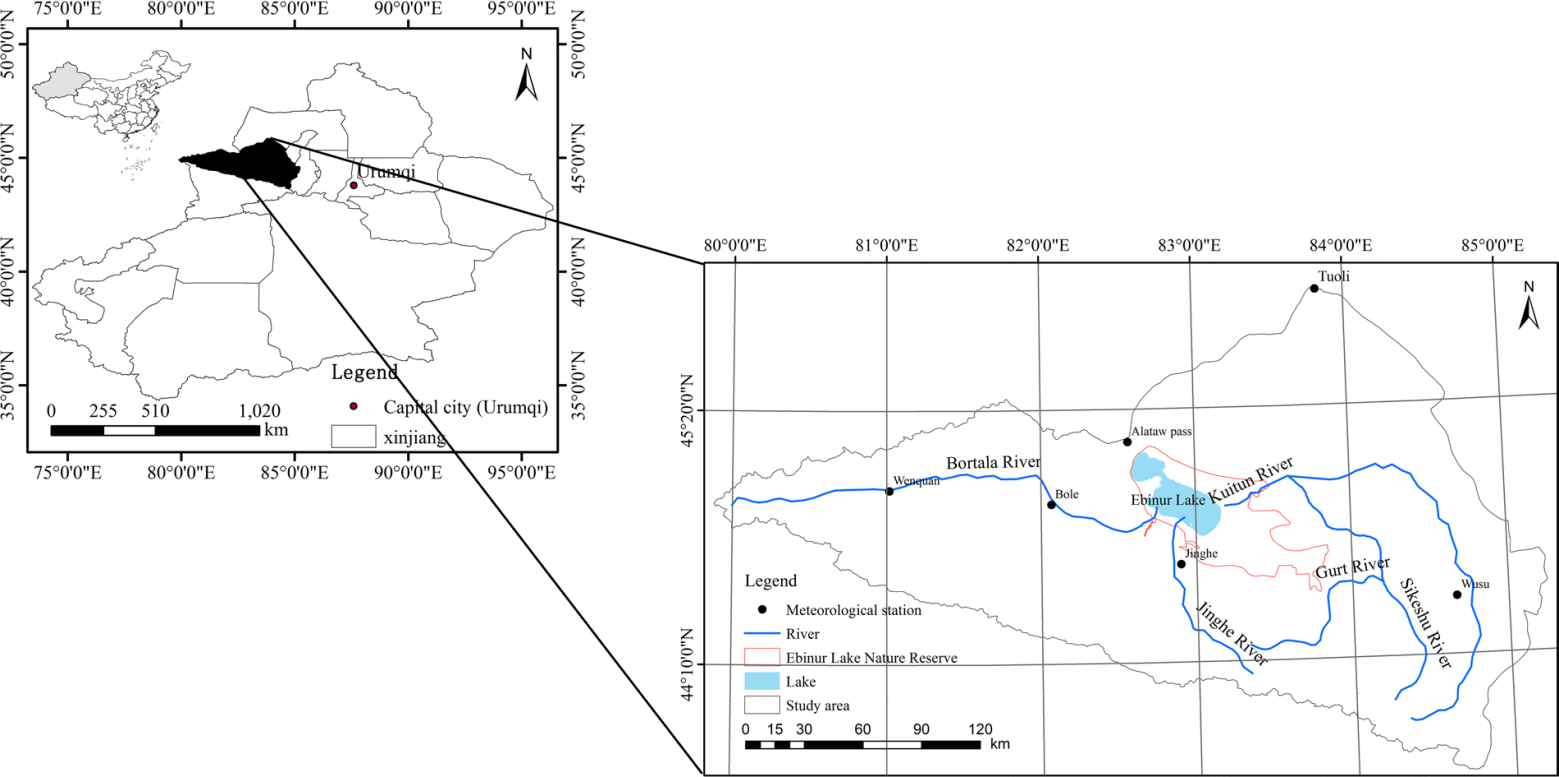
Geographical location of the Ebinur Lake watershed.

The Ebinur Lake watershed (43°38′–45°52′N, 79°53′–85°02′E) is a typical arid inland river watershed, located in the north-western region of Xinjiang Uygur Autonomous Region (XUAR) (Fig. 10); the watershed borders the northern slope of the Tianshan Mountains, is southwest of the Junggar Basin and west of the Bortala Valley. It belongs to the typical continental arid climate zone, with precipitation of only 100–200 mm, and Ebinur Lake is the largest salt lake in XUAR^52^. There are specific climate characteristics due to the complex landscape pattern, which has a mountain-oasis-desert ecosystem^53^. The overall ecological environment is highly vulnerable. The unique natural geographical factors determine the ecological environment in the watershed, and the ecological environment is extremely fragile; for example, desertification and salinization are serious, and sandstorms are frequent^54^. The vegetation coverage and lake area health environment of the Ebinur Lake watershed are also related to the industrial and agricultural production and economic sustainable development of the northern slope of the Tian Mountains, affecting the smooth flow of international transportation of the Eurasian Continental Bridge. The deterioration of the ecological environment caused by the shrinking of Ebinur Lake in the past 50 years, the extent of its ecological hazards, and the serious consequences make the ecological security of Ebinur Lake an urgent issue related to the overall social and economic development of Xinjiang^55^. The vegetation and lake area have changed due to climate change and anthropogenic activities, and these results have been reported in previous studies. In addition, the Kuitun River has become a seasonal river; furthermore, only the Bortala River and Jinghe River flow into the lake^56,57^.

## Experimental Design

### Normalized difference vegetation index data

The NDVI dataset is obtained from the US National Aeronautics and Space Administration (NASA MODIS Vegetation Index product data MOD13Q1 with a spatial resolution of 250 m and a temporal resolution of 16 d during growing seasons of 2000 to 2015; these data were downloaded from (http://ladsweb.nascom.nasa.gov). The dataset of MOD13Q1 was pre-processed with regard to radiation correction, atmospheric correction, and coordinate transformation, and the images are available for the vegetation coverage calculation with cloud removal and removal of bad lines^58^.

### Lake water body extraction and area variation detection

The lake water body data were derived from Landsat satellite imagery datasets. The datasets in this paper were mainly obtained from Landsat 5, 7 and 8 series satellites^59^. The remote sensing software ENVI and ArcGIS were utilized to extract the lake water body area and analyse the dynamic variation in the lake water body area during the research period.

The spectral water index was derived from different arithmetic operation and spectral properties, and the results showed heterogeneity in different media surfaces. Thus, appropriate thresholds of the indices were utilized to separate water bodies from other land-cover characteristics based on the spectral characteristics, in which water obviously absorbs energy at near-infrared (NIR) and shortwave-infrared (SWIR) wavelengths. The optical spectral water indices, such as the normalized difference water index (NDWI) and modified normalized difference water index (MNDWI), can maximize the spectral features used to distinguish water and land surface. According to the reports of Han *et al*., research on the dynamic variation of Qinghai Lake and the use of the MNDWI to extract lake information can satisfy the accuracy requirement^60^; additionally, Tian *et al*. indicated that for Poyang Lake, which is surrounded by urban and dense built-up regions, the MNDWI showed the most accuracy in terms of extracting the water body from the urban water region^61^. The MNDWI was determined to be more suitable for distinguishing urban buildings and water areas in the SWIR band spectral information. However, in the research reports of Zhang *et al*., the use of the spectral water MNDWI for water extraction in Ebinur Lake may lead to a misclassification between the water and surrounding dry lakebed because the MNDWI tends to weaken the effect of the mountain shadows^62^; this weakening may result in an overestimation of the water area. This result indicated that the NDWI might be a more appropriate spectral index for water extraction in natural and agricultural environments in arid and semi-arid regions. In this paper, the NDWI was utilized for water extraction, and this index has been widely used globally^63–65^. This index considers the format of the NDVI, as follows:

1 $${\rm{NDWI}}=\frac{{\rho }_{GREEN}-{\rho }_{NIR}}{{\rho }_{GREEN}+{\rho }_{NIR}}$$

where NDWI is the spectral water index; *ρ*_*GREEN*_ is the reflectivity in the green band; and *ρ*_*NIR*_ is the reflectivity in the NIR band. By setting reasonable threshold value, counting the number of pixels within the threshold, and calculates the area value of the lake^31^.

The dynamic degree of the lake water surface area characterizes the variation ratio of the lake surface area in relative observation periods. This index quantifies the dynamic variation rate of the shrinkage and expansion of the lake^66^. In this study, lake dynamics are calculated to characterize the annual variation of lakes as follows:

2 $$K=\frac{{U}_{b}-{U}_{a}}{{U}_{a}}\times \frac{1}{T}\times 100 \% $$

where *U*_*a*_ and *U*_*b*_ are the areas at the beginning and the end of the period, respectively; *T* is the study period; and *K* is the value of the annual rate of area dynamics.

### Accuracy evaluation and validation

Based on previous research results combined with field investigations in the study area, evaluating the accuracy of the water body extraction is necessary. Feyisa *et al*. proposed a method for automatically extracting water bodies and used the maximum likelihood method of supervised classification to verify the accuracy of the water body extraction with good precision^67^. To accurately evaluate the accuracy of the NDWI model in extracting the lake water area, the water area extracted from Landsat-5,7,8 using visual interpretation was regarded as the ground-truth lake water area, and a quantitative statistical analysis method was used to examine the classification accuracy of the NDWI model^68^. Based on the ground-truth water areas from Landsat (there images of September 8^th^, 2000, September 13^th^, 2008 and September 17^th^, 2015), the accuracy of the NDWI category was assessed by utilizing the maximum likelihood classification confusion matrix method. Through the value of the kappa coefficient, the producer and user accuracy and commission and omission errors were used to evaluate the overall accuracy^69^.

### Meteorological and Hydrological datasets

The meteorological dataset was derived from the China National Meteorological Information Center (http://data.cma.cn/) for the period from 1960 to 2015. The observed parameters include the monthly average temperature, precipitation and sunshine duration. All data were selected after a rigorous quality inspection and control. The missing meteorological data of each station were filled using the method of linear interpolation. The runoff data of the Bortala River and Jinghe River and the amount of water flow into the lake were acquired from the Hydrological Department in XUAR, China. In this paper, the sub-interval of 2000–2015 was the research period associated with the NDVI change.

### Vegetation and LUCC datasets

In this paper, we utilized a vegetation ecological map dataset with a scale of 1:1,000,000, and this map dataset was derived from the Data Center for Resources and Environmental Sciences, Chinese Academy of Sciences (RESDC); these maps were used to analyse the correlation between different terrestrial ecosystems and droughts at multiple temporal scales.

## Methods

### Trend analysis and the mann-kendall trend test

The Theil-Sen median trend analysis, non-parametric Mann-Kendall test and Hurst index were used to analyse the trend of climate and vegetation coverage. Non-parametric tests make no assumptions about the distribution of the data and are widely used for trend analysis of spatiotemporal variations^51,70,71^. The Sen trend analysis was used to analyze the spatial distribution characteristics of NDVI series;and the formula for Sen trend analysis is calculated as follows^72^:

3 $$Slope=Median\left[\frac{(NDV{I}_{j}-NDV{I}_{i})}{(j-i)}\right],\forall 1 < i < j < n$$

where *j* and *i* refers to time series indexes, *N*_*DVIj*_ and *NDVI*_*i*_ are the NDVI values of time series in pixels *j* and *i*. When Slope > 0, NDVI refers to an upward trend; when Slope < 0, NDVI refers to a downward trend.

The Mann-Kendall trend test does not require a specific sample distribution, and this condition is more suitable for an array of data and is useful for detecting monotonic trends^73,74^. The Mann-Kendall test is widely used for trend analysis in climatic studies^75,76^. Thus, in this study, the Mann-Kendall test was utilized to evaluate the trend of the NDVI based on pixels. The calculation process is as follows:

4 $${Z}_{c}=\left\{\begin{array}{ll}\frac{S-1}{\sqrt{var(s)}},\, & S > 0\\ 0\, & S=0\\ \frac{S-1}{\sqrt{var(s)}},\, & S < 0\end{array}\right.$$

5 $$S=\mathop{\sum }\limits_{i=1}^{n-1}\mathop{\sum }\limits_{k=i+1}^{n}sign(NDV{I}_{k}-NDV{I}_{i})$$

6 $${\rm{sign}}(NDV{I}_{k}-NDV{I}_{i})=\left\{\begin{array}{cc}1 & NDV{I}_{k}-NDV{I}_{i} > 0\\ 0 & NDV{I}_{k}-NDV{I}_{i}=0\\ -1 & NDV{I}_{k}-NDV{I}_{i} < 0\end{array}\right.$$

where *NDVI*_*k*_ and *NDVI*_*i*_ are the subsamples of the time-series datasets; *n* is the length of the series dataset; *S* is the test statistic; *Sign* is the sign function; Due to the difference in the *n* values of time series period, the appropriate statistical test for judging significance differs. Mann and Kendall *et al*. indicated that when n ≥ 8, the statistic *S* roughtly obeys the normal distribution^77,78^, otherwise, when *n* < 8, the test statistic *S* is used directly for a bilateral trend test; and if *S* > 0, *S* = 0 or *S* < 0 refers to an upward trend, non-trend and downward trend, respectively. In this study, the time series was of length *n* > 10, thus, the significant statistic test *Z* was untilized to examine the trend. Under the given significance level of α = 0.05, which corresponds to the value of *Z* = ±1.96 and implies a significant change when $|Z| > {U}_{1-\alpha /2}$. Additionally, $\pm {Z}_{1-\alpha /2}$ is the standard normal deviation. |*Z*| > 1.96 illustrates the time series is significant at the level α = 0.05, and |Z| < 1.96 illustrates the time series is not significant at the level α = 0.05^79^.

### Hurst index

The Hurst exponent has been widely used in economics, hydrology and climatology to quantitatively analyse the sustainability of a series dataset^80^, and this index does not make assumptions about the statistical restrictions. In addition, the Hurst index was applied early in the hydrological predictions of the Nile River. In this study, the R/S analysis is used to calculate the H index as follows:

The cumulative deviation is calculated as follows:

7 $${X}_{(t,\tau )}=\mathop{\sum }\limits_{t=1}^{t}({X}_{(t)}-\overline{{X}_{(\tau )}})\,1\le t\le \tau $$

The extreme deviation sequence is formulated as follows:

8 $${R}_{(\tau )}=\,{\rm{\max }}\,{X}_{(t,\tau )}-\,{\rm{\min }}\,{X}_{(t,\tau )}\,\tau =1,2,\cdots ,{\rm{n}};1\le {\rm{t}}\le {\rm{\tau }}$$

The standard deviation sequence is calculated as follows:

9 $${S}_{(\tau )}={\left[\frac{1}{\tau }\mathop{\sum }\limits_{t=1}^{\tau }{({X}_{(t)}-{X}_{\tau })}^{2}\right]}^{\frac{1}{2}}\,\tau =1,2,\cdots ,n$$

The Hurst index is calculated as follows:

10 $$\frac{R(\tau )}{S(\tau )}={(c\tau )}^{H}$$

According to the formula, the Hurst exponent is derived by the least squares method and ranges from 0 to 1, which reveals the fractal characteristics of the time series. When H = 0.5, the trend shows no obvious correlation. When 0 < H < 0.5, the trend implies an opposite variation from that of the past in the future, and when H is smaller than 0, the anti-sustainability value is stronger. However, when H > 0.5, the trend implies the value is consistent with that in the past and is related to the magnitude of H.

### SPEI calculation

The newly developed SPEI was used to characterize the drought evolution in the Ebinur Lake basin from 1960to2015. Considering the availability of meteorological datasets, the monthly difference in precipitation and potential evapotranspiration was calculated for the SPEI using the Thornthwaite method^81^. With the calculation of the monthly values for precipitation (P) and potential evapotranspiration (PET), the difference between P and PET for the ith month is calculated as follows^15^:

11 $$PE{T}_{i}=\left(\frac{N}{12}\right)\left(\frac{NDM}{30}\right){\left(\frac{10T}{I}\right)}^{m}$$

12 $${\rm{i}}={(T/5)}^{1.514}$$

13 $$m=6.75\times {10}^{-7}{I}^{3}-7.71\times {10}^{-5}{I}^{2}+1.79\times {10}^{-2}I+0.492$$

where *T* is the monthly mean temperature (°C), *N* is the maximum number of sun hours, *NDM* is the number of days in the month, and *I* is the heat index. The heat index is calculated as the sum of 12 monthly index values *i*; the latter is calculated by the mean monthly temperature; and m is a coefficient that depends on *I*.

14 $${D}_{i}={P}_{i}-PE{T}_{i}$$

The calculated D values are aggregated at various time scales as follows:

15 $${D}_{n}^{k}=\mathop{\sum }\limits_{i=0}^{k-1}({P}_{n-i}-PE{T}_{n-i}),n\ge k$$

where *P*_*i*_ is *i*th month precipitation, *PET*_*i*_ is the ith month potential evapotranspiration calculated by the Thornthwaite method, and *k* and *n* are the timescale of the aggregation and the calculation number, respectively. ${D}_{n}^{k}$ is based on both the *n*^*th*^ climatic water balance and the water balance for the preceding *k*−1 months. For instance, the 3-month SPEI is constructed by the sum of the *D* values from two previous months to the current month. The criteria for drought classification based on SPEI values are defined^37^. In this study, the multiple temporal scale correlations of SPEI-1, SPEI-3, SPEI-6, SPEI-12 and SPEI-24 between the vegetation dynamics and drought were calculated. Table 2 shows the standard for using the SPEI to classify drought. Both the SPEI and SPI standardized algorithms are similar; thus, the SPEI is classified with reference to the classification standard of the SPI drought category^82^ (Table 2).

**Table 2:** Drought classification for the SPEI.

Drought class	SPEI value
Extreme wet	SPEI > 2.0
Severe wet	1.5 < SPEI ≤ 2.0
Moderate wet	1.0 < SPEI ≤ 1.5
Normal	−1.0 < SPEI ≤ 1.0
Moderate drought	−1.5 < SPEI ≤ −1.0
Severe drought	−2.0 < SPEI ≤ −1.5
Extreme drought	SPEI ≤ −2

### Correlation analysis

In this study, Pearson correlation analysis was used to analyse the correlation between the NDVI series and the SPEI at different temporal scales (1, 3, 6, 12, and 24 months) from April to October. The calculation method is as follows:

16 $$\begin{array}{c}{R}_{i,j}=corr(NDV{I}_{i},SPE{I}_{i,j}),4\le i\le 10,j=1,3,6,12,24\\ {R}_{max}=ma{x}_{4\le i10,j=1,3,6,12,24}\end{array}$$

where corr represents the correlation between the NDVI series for each month and the SPEI; *i* represents the *i*th month, ranging from April to October; *j* represents the different values of the drought scale (i.e., 1, 3, 6, 12, and 24 months); *NDVI*_*i*_ and *SPEI*_*i*_ are the ith month of the NDVI series and the *i*th month of the SPEI of different time scales for *j* month, respectively; and *R*_*max*_ represents the maximum correlation coefficient between *NDVI*_*i*_ and *SPEI*_*i,j*_. Thus, there were 35 *R*_*i,j*_ correlation coefficients for each pixel. In this study, the observations of the NDVI series length span 15 years; thus, the correlation coefficient values of 0.51 and 0.64 correspond to the 5% and 1% significance levels, respectively. As known, the natural plant phenomena and environments change periodically, and these changes eliminate the influence of phenology on the correlation analysis results. Only the maximum value (*R*_*max*_) was derived from the 35 correlation coefficients for each month of the different temporal scales in order to assess the impact of drought on vegetation NDVI in the growing season, spring, summer, autumn and winter^29^.
